# Plasma Energy-Balance Metabolites Discriminate Asymptomatic Patients with Peripheral Artery Disease

**DOI:** 10.1155/2018/2760272

**Published:** 2018-09-20

**Authors:** Anna Hernández-Aguilera, Salvador Fernández-Arroyo, Noemí Cabre, Fedra Luciano-Mateo, Gerard Baiges-Gaya, Montserrat Fibla, Vicente Martín-Paredero, Javier A. Menendez, Jordi Camps, Jorge Joven

**Affiliations:** ^1^Unitat de Recerca Biomèdica, Hospital Universitari Sant Joan, Institut d'Investigació Sanitària Pere Virgili, C. Sant Joan s/n, 43201 Reus, Spain; ^2^Universitat Rovira i Virgili, C. Escorxador s/n, 43003 Tarragona, Spain; ^3^Department of Vascular Surgery, Hospital Universitari Joan XXIII, C/ Dr. Mallafré Guasch 4, 43005 Tarragona, Spain; ^4^Program Against Cancer Therapeutic Resistance (ProCURE), Metabolism and Cancer Group, Catalan Institute of Oncology, Girona, Spain; ^5^Girona Biomedical Research Institute (IDIBGI), Girona, Spain; ^6^The Campus of International Excellence Southern Catalonia, Tarragona, Spain

## Abstract

Peripheral artery disease (PAD) is a common disease affecting 20–25% of population over 60 years old. Early diagnosis is difficult because symptoms only become evident in advanced stages of the disease. Inflammation, impaired metabolism, and mitochondrial dysfunction predispose to PAD, which is normally associated with other highly prevalent and related conditions, such as diabetes, dyslipidemia, and hypertension. We have measured energy-balance-associated metabolite concentrations in the plasma of PAD patients segregated by the severity of the disease and in plasma of healthy volunteers using a quantitative and targeted metabolomic approach. We found relevant associations between several metabolites (3-hydroxybutirate, aconitate, (iso)citrate, glutamate, and serine) with markers of oxidative stress and inflammation. Metabolomic profiling also revealed that (iso)citrate and glutamate are metabolites with high ability to discriminate between healthy participants and PAD patients without symptoms. Collectively, our data suggest that metabolomics provide significant information on the pathogenesis of PAD and useful biomarkers for the diagnosis and assessment of progression.

## 1. Introduction

The prevalence of peripheral artery disease (PAD) is now higher than 20% in population over 60 years, and affected patients have a several-fold increased risk of all-cause mortality compared to people without the disease [1]. Age, hypertension, hypercholesterolemia, diabetes, and smoking are recognized risk factors, but the disease progresses silently for decades, with the consequence that if appropriate, effective measures are applied too late, or not implemented at all, will result in atherosclerosis affecting wide portions of the arteries in the lower extremities [2]. Tissue ischemia is a common finding in PAD patients, and an increasing body of evidence supports the notion that inflammation plays an important role in the pathogenesis of this disease, linking oxidative stress, metabolic adaptation, and immunity [3–6]. Consequently, the combined action of abnormal mitochondrial function, increased production of reactive oxygen species, impaired energy metabolism, and the subsequent inflammatory response complicates vascular remodeling, perfusion recovery, and atherosclerosis [7–10].

One of the main challenges clinicians face is early diagnosis of PAD (i.e., during the asymptomatic stages), and many biomarker candidates have been proposed with limited success [11–13]. The emerging metabolomic approaches are providing new clues in understanding atherosclerosis-related cardiovascular disease [14–20], but efforts on PAD have been scarce. Previous studies from our group suggested that serum paraoxonase-1 (PON1) and the chemokine (C-C) motif ligand 2 (CCL2) might be useful biomarkers of PAD [21, 22]: PON1 being a scavenger of excessive reactive oxygen species [23] and CCL2 an inducer of monocyte migration and differentiation into macrophages [24]. As mitochondrial dysfunction and inflammation result in disrupted metabolism [25, 26], we report here that metabolomic profiling of plasma may be useful for identifying patients at increased risk of PAD and that energy-balance metabolites are associated with inflammation and oxidation in these patients. Our results might suggest new biomarkers and therapeutic targets.

## 2. Materials and Methods

### 2.1. Participants and Study Design

We performed an observational, cross-sectional study in 201 men with clinically diagnosed PAD attending our vascular surgery department between 2010 and 2015. Patients were classified according to the Fontaine classification in nonsymptomatic stage (grade I), intermittent claudication (grade II), rest pain (grade III), and tissue damage, and necrosis (grade IV) [27]. Diagnostic criteria involved ankle-brachial index (ABI), noninvasive imaging techniques (computerized tomography scan or magnetic resonance imaging), and arteriography when indicated. The exclusion criteria were the presence of acute ischemia, signs of infection, renal failure, liver disease, cancer, or autoimmune disease. Clinical data and laboratory variables were obtained from patients' clinical records. For comparisons, we used biobanked samples (*n* = 48) from healthy, age-matched men and normal ABI values, whose details have been previously described [28]. Plasma and serum samples from all participants were collected and stored at −80°C until use. Procedures were approved by the Comité d'Ètica d'Investigació Clínica of Hospital Universitari de Sant Joan (EPINOLS/12-03-09/3proj6 and INFLAMET/15-04-30/4proj6), and written informed consent was obtained from all participants.

### 2.2. Metabolomic Analyses

To detect and quantify metabolites of energy metabolism, we used a previously described method [6]. Briefly, metabolites from plasma (100 *μ*l) were extracted using 400 *μ*l of methanol/water (8 : 2, *v*/*v*) and proteins were precipitated for two hours at −20°C. After centrifugation at 14,000 rpm for 10 minutes at 4°C, the supernatant was collected and dried under N_2_ flow. Metabolites were then derivatized with methoxyamine in pyridine (40 mg/ml) and N-methyl-N-(trimethylsilyl)-trifluoroacetamide and injected into a gas chromatograph coupled with a quadrupole time-of-flight mass spectrometer by an electron impact source. Metabolites were detected and quantified with the use of proper calibration curves.

### 2.3. Biochemical Analyses

The true physiological substrates for PON1 have not been yet identified. Since PON1 has lactonase and esterase activities [23], we opted to analyze the catalytic activity of PON1 using two different substrates: paraoxon (an ester) and 5-thiobutyl butyrolactone (TBBL; a synthetic lactone). Serum PON1 paraoxonase activity was determined as the rate of hydrolysis of paraoxon at 410 nm and 37°C in a 0.05 mM glycine buffer, pH 10.5 with 1 mM CaCl_2_. Activities were expressed as U/l (1 U = 1 *μ*mol of paraoxon hydrolyzed per minute). Serum PON1 lactonase activity was measured in an assay containing 1 mM CaCl_2_, 0.25 mM 5-thiobutyl butyrolactone, and 0.5 mM 5,5′-dithio-bis-2-nitrobenzoic acid (DTNB) in 0.05 mM Tris-HCl buffer, pH = 8.0. The change in absorbance was monitored at 412 nm. Activities were expressed as U/l (1 U = 1 mmol of TBBL hydrolyzed per minute) [29]. Serum PON1 concentrations were determined by an in-house enzyme-linked immunosorbent assay (ELISA) with a rabbit polyclonal antibody generated against the synthetic peptide CRNHQSSYQTRLNALREVQ which is sequence specific for mature PON1 [30]. CCL2 metabolites were measured by ELISA (PeproTech, London, UK). Serum lipid, liver profiles, and glucose concentrations were analyzed by standard tests in a Roche Modular Analytics P800 system. Blood count was analyzed in a Roche Sysmex XT-1800i counter (Roche Diagnostics, Basel, Switzerland).

### 2.4. Statistical Analysis

Differences between groups were assessed by Student's *t*-test (parametric) or Mann–Whitney *U* test (nonparametric). Correlations between variables were assessed by Spearman's rho test. The relative magnitude of observed changes was evaluated using random forest analysis [31]. Receiver operating characteristic (ROC) curves were used to assess the diagnostic accuracy of the measured variables. This analysis represents plots of all the sensitivity/specificity pairs resulting from varying decision thresholds. Sensitivity (or true-positive rate) is the proportion of the sample correctly identified as having a specific disease. Specificity (or true-negative rate) is the proportion of the subjects correctly identified as not having a specific disease. False-positive rate is calculated as 1 − specificity. The area under the curve (AUC) and 95% confidence interval (CI) were calculated. The AUC represents the ability of the test to correctly classify patients according to the investigated alteration. The values of AUC can range between 1 (perfect test) and 0.5 (worthless test) [32]. Statistical analyses were performed with SPSS 22.0 (IBM Corp., Chicago, IL, USA). MetaboAnalyst 3.0 (http://www.metaboanalyst.ca/) was used to generate scores/loading plots and random forest analyses.

## 3. Results

### 3.1. Participants' Characteristics

Clinical characteristics and biochemical variables of PAD patients and the control group are shown in Table 1. PAD patients had a mildly higher BMI than those in the control group, and they often had diabetes, hypertension, or dyslipidemia. Serum glucose and triglyceride concentrations were higher in patients, while cholesterol was lower, which is probably the result of medication.

### 3.2. Alterations in Energy-Balance-Associated Metabolites

**Figure 1:**
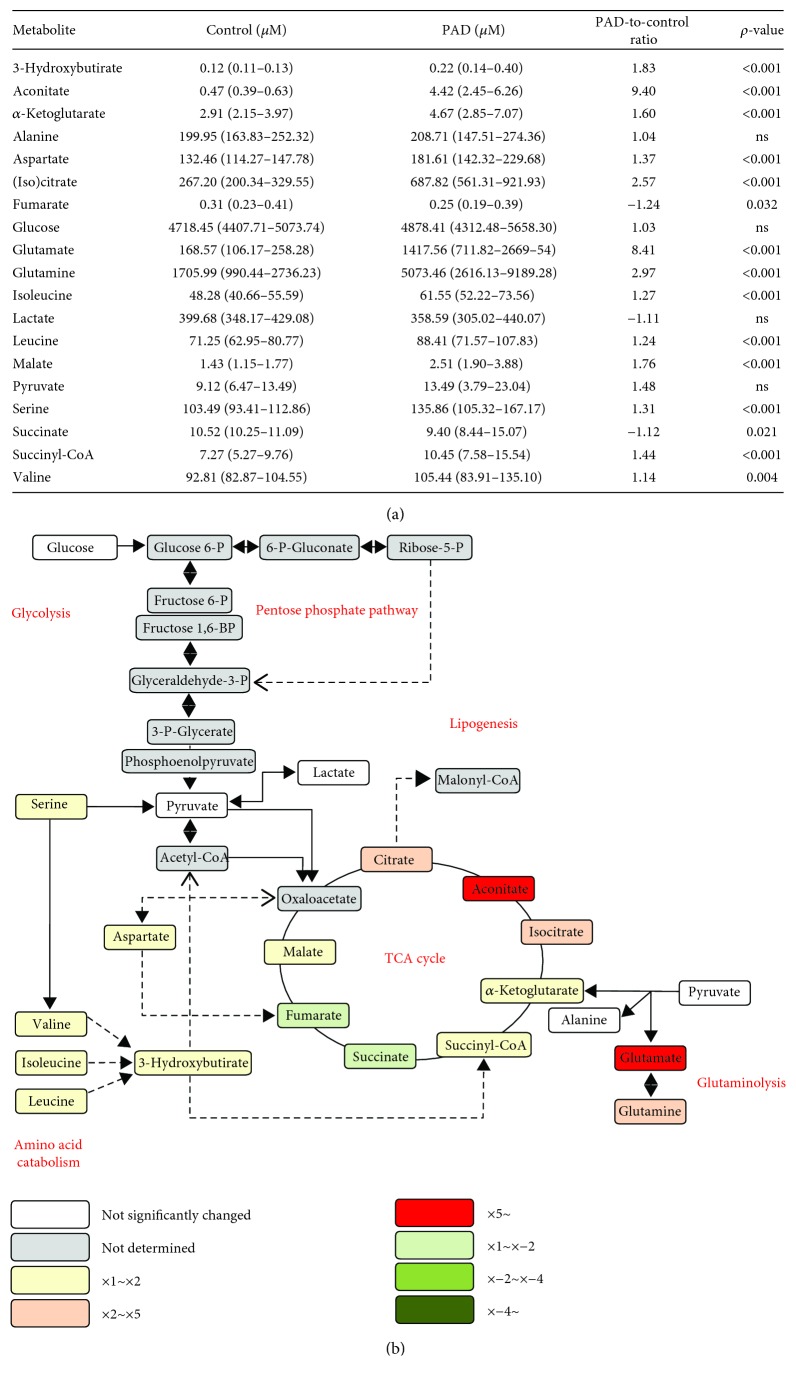


The plasma concentrations of most energy metabolism intermediates were significantly higher in PAD patients than those in the control group, with the exceptions of fumarate, lactate, and succinate, that were decreased (Figure 1(a)). Glutaminolysis was disrupted in PAD, as shown by glutamate and glutamine increases. Moreover, reactions involving amino acid metabolism seemed to be inhibited in PAD, as serine, valine, isoleucine, and leucine concentrations were increased. TCA cycle was strongly disturbed since (iso)citrate, aconitate, *α*-ketoglutarate, succinyl-CoA, and malate concentrations were increased, and fumarate and succinate concentrations were decreased in PAD (Figure 1(b)).

### 3.3. Relationships between Energy-Balance-Associated Metabolites, PON1 and CCL2

We observed significant inverse correlations between metabolites and PON1-related variables and direct correlations between metabolites and CCL2. The strongest correlations were observed between serum PON1 concentration and PON1 lactonase activity with 3-hydroxybutirate, aconitate, (iso)citrate, glutamate, and serine (Table 2).

### 3.4. Metabolites Are Linked to PAD Comorbidities, Age, and BMI

**Figure 2:**
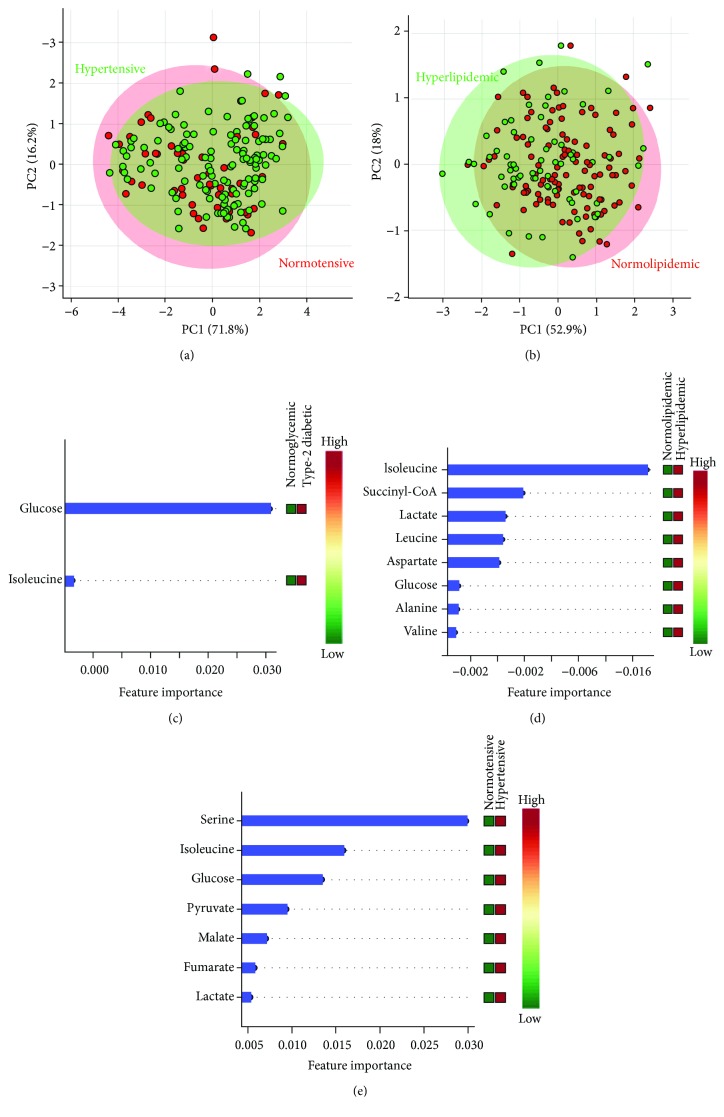


**Figure 3:**
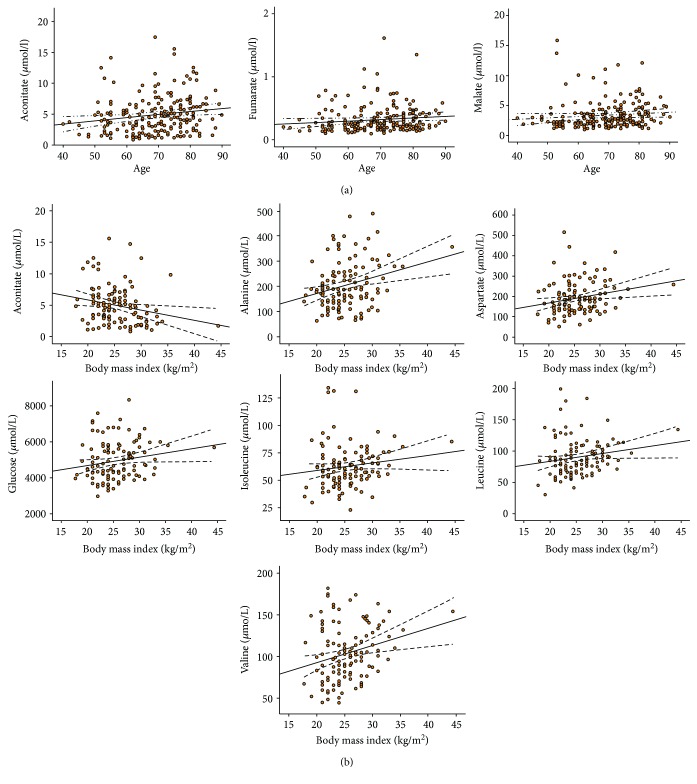


Univariate analyses confirmed that many metabolites were associated with diabetes, hypertension, or dyslipidemia (Tables 3–5), while multivariate analyses (principal component analyses) revealed that the combination of these metabolites was not able to separate groups regarding hypertension (Figure 2(a)) and dyslipidemia (Figure 2(b)). Random forest analysis showed that glucose and isoleucine were associated with diabetes in PAD patients and glucose (as expected) had the highest discriminant capacity (Figure 2(c)). Hyperlipidemic and normolipemic patients showed significant differences in alanine, aspartate, glucose, isoleucine, lactate, leucine, succinyl-CoA, and valine concentrations and, among them, isoleucine had the highest discriminant capacity (Figure 2(d)). Fumarate, glucose, isoleucine, lactate, malate, serine, and pyruvate were associated with hypertension in PAD patients, and serine was the metabolite with the best discriminant capacity (Figure 2(e)). Aconitate, fumarate, and malate were associated with age, and aconitate, alanine, aspartate, glucose, isoleucine, leucine, and valine were correlated with BMI (Table 6 and Figure 3). All these metabolites were excluded for further analysis as candidates for PAD biomarker in order to avoid the influence of confounding factors.

### 3.5. Metabolic Biomarkers of PAD

**Figure 4:**
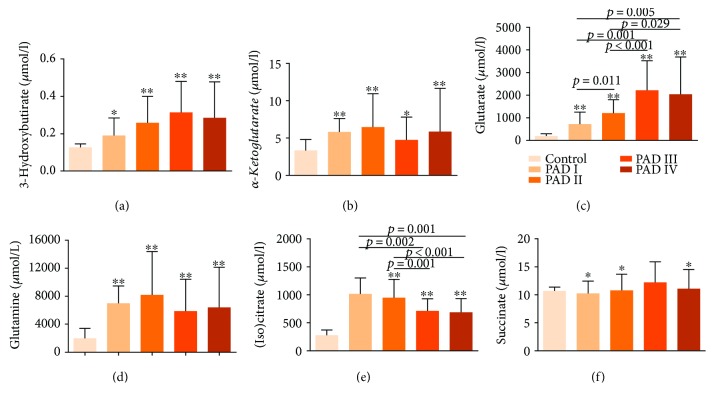
^∗^ *p* < 0.05 and ^∗∗^*p* < 0.001 for the control samples.

**Figure 5:**
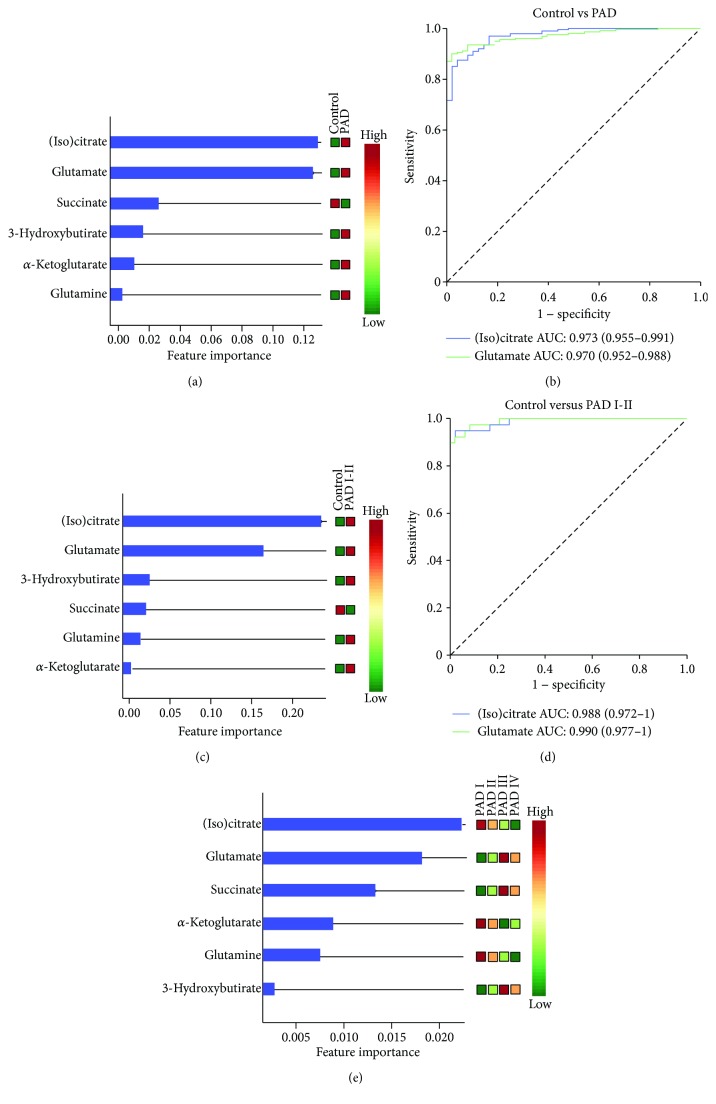


Metabolites included in further analysis were (iso)citrate, glutamate, succinate, 3-hydroxybutirate, *α*-ketoglutarate, and glutamine. From them, (iso)citrate and glutamate were those showing the greatest differences between patients and controls and between the different patient groups according to the Fontaine scale (Figure 4). To evaluate the capacity of these metabolites to discriminate between groups, we performed random forest analyses and ROC curves confirming that (iso)citrate and glutamate were the most powerful metabolites to separate the healthy individuals from PAD patients (Figures 5(a) and 5(b)), and the healthy individuals from asymptomatic or nearly asymptomatic patients (grades I and II), with AUC > 0.95 (Figures 5(c) and 5(d)). They were also useful to discriminate between the different subgroups of patients (Figure 5(e)).

## 4. Discussion

Energy-balance-associated metabolites are related to oxidative stress and inflammation. We found in PAD patients significant alterations in energy metabolism, particularly evident through the citrate-aconitate-(iso)citrate conversions. The mitochondrial enzymes involved in these reactions are (iso)citrate dehydrogenase (IDH2) and aconitase 2 (ACO2). Both enzymes are crucial for normal mitochondrial function [33]. In mice, decreased IDH2 expression contributes to atherosclerosis progression by increasing oxidative stress [34]. An oxidative environment inactivates aconitase, which in turn undergoes age-dependent oxidative modification. Whether IDH2 and ACO2 may be the cause or consequence of mitochondrial dysfunction in PAD requires further studies [8].

Mitochondrial dysfunction, oxidative stress, and inflammation are closely related [24, 25]. The present study shows the existence of significant inverse correlations between various metabolites and PON1-related variables and direct correlations with CCL2. These correlations are stronger for PON1 concentration and lactonase activity. This may be due to the observation that PON1 is located (among other cellular organelles) in the membranes of the mitochondria, protecting them from oxidative stress [25]. Therefore, alterations in the TCA cycle may directly affect PON1. The correlations between metabolites and paraoxonase activity are weaker than those found for lactonase activity, but this is probably due to the differential impact of genetic polymorphisms [23]. Metabolites showing the strongest and more consistent correlations with PON1 and CCl2 were 3-hydroxybutirate, aconitate, (iso)citrate, glutamate, and serine. 3-Hydroxybutirate is a ketone body, and studies on the effects of ketones on oxidative stress and inflammation are contradictory. For example, it has been reported that these compounds inhibit mitochondrial production of reactive oxygen species in rat and mice neurons [35–37] while they activate NF-*κ*B, upregulate NADPH oxidase, elevate oxidative stress, and induce the expression of proinflammatory cytokines in endothelial cells and hepatocytes [38, 39]. Our results are in agreement with this latter possibility. It has been suggested that these contradictory findings reflect tissue-specific differences because the source of reactive oxygen species in neurons differ from that in nonneuronal cells [35]. Aconitate is the precursor of itaconate, and this metabolite regulates metabolic remodeling and mitochondrial respiration in inflammatory macrophages [40]. There is little information on the associations of (iso)citrate, glutamate, and serine with oxidative stress and inflammation, but studies suggest that glutamate is pro-oxidant and proinflammatory [41] while (iso)citrate and serine may elicit the opposite effect [42, 43].

Energy-balance-associated metabolites might be considered as PAD biomarkers. Early diagnosis is important in these patients because preventive treatment has potential benefits in the progression of PAD. Current biomarkers are indeed risk factors [11, 13], and dyslipidemia, hypertension, or diabetes plays independent roles in atherogenesis. In our population, approximately 80% of patients had one or more of these complications. Their effect on metabolite concentrations is unknown but probably multifactorial. For instance, branched-chain amino acids (BCAA) were influenced by hypertension, diabetes, and dyslipidemia confirming previous associations [44–47]. We therefore discarded metabolites influenced by confounding variables, and we found six candidates with statistically significant differences in concentration between the control group and PAD patients: 3-hydroxybutyrate, *α*-ketoglutarate, glutamate, glutamine, (iso)citrate, and succinate. These candidates were useful to distinguish between PAD patients and the control group and also to discriminate between different clinical stages. The conversion of (iso)citrate to *α*-ketoglutarate is mediated by IDH2, and increased concentrations of this metabolite have been associated with a worse cardiovascular prognosis [48]. Interestingly, glutamate, another metabolite with a good discriminant capacity, is the substrate for many enzymes located in the mitochondria [49] and plays an important role in the mechanical function of the ischemic myocardium [50]. Understanding glutamate overproduction in the blood of patients with atherosclerosis requires further research. Our more interesting finding indicates that (iso)citrate and glutamate may discriminate healthy participants from PAD patients in the asymptomatic or early symptomatic stages (Fontaine grades I and II). Simple measurements may then provide clinical tools to assess patients at risk but without symptoms. Larger studies using sensitive metabolomic techniques are warranted to confirm these findings and to identify specific metabolic pathways associated with increased risk of PAD.

## 5. Conclusion

Our metabolomic approach supports a relevant association of plasma concentrations of energy-balance-associated metabolites with oxidative stress and inflammation and reveals (iso)citrate and glutamate as candidate biomarkers for discriminating PAD patients without symptomatic disease.

## Figures and Tables

**Table 1:** Clinical characteristics, blood count, and biochemical characteristics of the control group and PAD patients.

	Control (*n* = 48)	PAD (*n* = 201)	*p* value
BMI (kg/m^2^)	24 (22.5–25.3)	25 (22.5–28)	0.021
Diabetes (%)	—	64.1	<0.001
Hypertension (%)	—	69.2	<0.001
Dyslipidemia	—	37.9	<0.001
Red blood cells, ×10^12^/l	4.9 (4.4–5.2)	4.16 (3.57–4.66)	<0.001
Hemoglobin, mmol/l	8.94 (8.32–9.43)	13.30 (11.50–14.90)	0.001
Leukocytes, ×10^9^/l	6.8 (5.4–8.2)	8.17 (6.50–10.22)	0.003
Platelets, ×10^9^/l	233 (205–273)	253 (200–329)	ns
Total cholesterol, mmol/l	4.85 (4.40–5.85)	3.90 (3.31–4.94)	<0.001
HDL cholesterol, mmol/l	1.34 (1.14–1.61)	0.96 (0.78–1.19)	<0.001
LDL cholesterol, mmol/l	2.82 (2.40–3.86)	2.26 (1.77–2.79)	<0.001
Triglycerides, mmol/l	0.90 (0.70–1.38)	1.99 (1.40–3.08)	<0.001
Glucose, mmol/l	4.70 (4.37–4.92)	5.61 (4.60–6.88)	<0.001
ALT, U/l	20 (13.5–24.9)	21.5 (15–34.8)	ns
AST, U/l	20 (17.7–24)	21 (16–32)	ns

BMI: Body mass index; HDL: high-density lipoprotein; LDL: low-density lipoprotein; ALT: alanine aminotransferase; AST: aspartate aminotransferase; ns: not significant. Nonparametric variables are shown as medians, and IQR are in parentheses. Qualitative variables are expressed as percentage of total participants.

**Table 2:** Spearman's correlation coefficients for PON1 concentration, PON1 paraoxonase activity, PON1 lactonase activity, CCL2 concentration, and related metabolites.

	PON1 concentration		Paraoxonase activity		Lactonase activity		CCL2 concentration	
	Spearman's rho	*p* value	Spearman's rho	*p* value	Spearman's rho	*p* value	Spearman's rho	*p* value
3-hydroxybutirate	−0.640	<0.001	−0.309	<0.001	−0.496	<0.001	0.241	0.009
*α*-Ketoglutarate	−0.021	ns	−0.183	0.029	−0.029	ns	−0.045	ns
Aconitate	−0.641	<0.001	−0.394	<0.001	−0.552	<0.001	0.345	<0.001
Alanine	0.153	ns	0.253	0.002	0.275	0.001	−0.155	ns
Aspartate	−0.241	0.004	−0.187	0.026	−0.349	<0.001	0.184	0.046
(Iso)citrate	−0.592	<0.001	−0.166	0.048	−0.381	<0.001	0.303	0.001
Fumarate	0.295	<0.001	−0.053	ns	0.166	0.048	−0.014	ns
Glucose	0.011	ns	0.109	ns	0.053	ns	−0.098	ns
Glutamate	−0.590	<0.001	−0.425	<0.001	−0.589	<0.001	.0171	ns
Glutamine	−0.263	0.002	−0.059	ns	−0.198	0.018	0.187	0.043
Isoleucine	−0.342	<0.001	−0.101	ns	−0.299	<0.001	0.167	ns
Lactate	0.277	0.001	0.086	ns	0.175	0.036	−0.104	ns
Leucine	−0.229	0.006	0.008	ns	−0.136	ns	0.079	ns
Malate	−0.356	<0.001	−0.240	0.004	−0.279	0.001	0.225	0.014
Pyruvate	0.356	<0.001	−0.034	ns	0.158	ns	−0.163	ns
Serine	−0.595	<0.001	−0.294	<0.001	−0.534	<0.001	0.295	0.001
Succinate	−0.355	<0.001	−0.194	0.021	−0.342	<0.001	0.110	ns
Succinyl-CoA	−0.223	0.008	−0.101	ns	−0.154	ns	0.025	ns
Valine	−0.035	ns	0.155	ns	0.076	ns	−0.009	ns

PON1: Paraoxonase 1; CCL2: chemokine (C-C) motif ligand 2; ns: not significant.

**Table 3:** Metabolite concentration in patients segregated according to the presence of diabetes.

Metabolite	Diabetes	
	No	Yes
3-hydroxybutirate	0.27 (0.13–0.39)	0.27 (0.14–0.40)
Aconitate	3.75 (2.38–6.50)	4.60 (2.67–6.28)
*α*-Ketoglutarate	4.24 (2.56–7.22)	4.13 (2.76–6.50)
Alanine	210.6 (147.5–266.3)	189.6 (139.0–271.4)
Aspartate	172.5 (133.3–215.8)	179.7 (143.0–225.1)
(Iso)citrate	721.7 (584.42–867.3)	665.6 (538.0–880.5)
Fumarate	0.26 (0.19–0.41)	0.24 (0.18–0.37)
Glucose	4546.0 (4104.0–5115.5)	4959.3 (4040.8–5804.9)^a^
Glutamate	1457.2 (743.0–2912.4)	1416.9 (671.7–2684.2)
Glutamine	5073.4 (3054.2–7754.5)	4742.5 (1842.3–8881.2)
Isoleucine	57.14 (47.71–63.99)	63.68 (52.83–75.20)^a^
Lactate	367.2 (283.9–423.6)	341.0 (297.1–441.6)
Leucine	85.07 (70.62–94.86)	86.68 (69.33–109.65)
Malate	2.38 (1.85–4.31)	2.45 (1.88–3.56)
Pyruvate	12.47 (3.64–22.87)	12.83 (3.55–21.89)
Serine	147.5 (101.7–167.4)	137.6 (109.4–169.5)
Succinate	9.57 (8.35–15.11)	9.70 (8.47–15.23)
Succinyl-CoA	10.14 (7.74–15.12)	10.46 (7.37–17.62)
Valine	102.0 (83.0–129.4)	104.1 (80.7–138.9)

^a^ *p* < 0.01.

**Table 4:** Metabolite concentrations in patients segregated according to the presence of hypertension.

Metabolite	Hypertension	
	No	Yes
3-hydroxybutirate	0.21 (0.14–0.38)	0.35 (0.14–0.42)
Aconitate	4.59 (2.54–6.65)	4.13 (2.60–6.60)
*α*-Ketoglutarate	4.71 (2.81–6.67)	3.70 (2.51–7.07)
Alanine	213.5 (152.7–273.7)	184.3 (137.3–255.1)
Aspartate	181.6 (141.4–239.5)	173.1 (137.3–200.4)
(Iso)citrate	721.8 (566.6–934.0)	654.2 (476.5–815.9)
Fumarate	0.27 (0.19–0.40)	0.22 (0.17–0.30)^a^
Glucose	4955.9 (4459.1–5679.9)	4418.1 (4134.8–5191.7)^b^
Glutamate	1335.8 (725.0–2628.5)	1786.6 (691.1–2891.7)
Glutamine	5083.3 (2842.0–9072.2)	4742.5 (1700.9–7003.8)
Isoleucine	63.52 (52.24–75.19)	57.88 (51.23–63.22)^a^
Lactate	373.5 (308.3–452.5)	332.3 (279.5–405.9)^a^
Leucine	90.17 (71.33–109.35)	84.54 (69.77–93.84)
Malate	2.79 (1.96–4.09)	2.23 (1.75–3.08)^a^
Pyruvate	13.86 (3.85–24.11)	8.78 (2.72–18.15)^a^
Serine	135.3 (106.1–162.1)	161.0 (103.6–172.0)^a^
Succinate	9.15 (8.42–15.04)	11.71 (8.49–15.47)
Succinyl-CoA	11.21 (7.72–16.31)	9.94 (7.52–14.25)
Valine	107.3 (86.6–136.8)	96.6 (78.8–134.8)

^a^ *p* < 0.05; ^b^*p* < 0.01.

**Table 5:** Metabolite concentrations in patients segregated according to the presence of dyslipidemia.

Metabolite	Dyslipidemia	
	No	Yes
3-hydroxybutirate	0.31 (0.16–0.40)	0.19 (0.13–0.419)
Aconitate	4.64 (2.91–6.61)	4.22 (2.36–6.16)
*α*-Ketoglutarate	3.99 (2.44–6.55)	5.05 (3.05-6.90)
Alanine	180.5 (125.1–247.5)	233.6 (171.4–302.8)^b^
Aspartate	168.6 (134.1–205.4)	191.7 (148.8–253.2)^a^
(Iso)citrate	678.6 (545.4–864.5)	712.3 (566.1–959.1)
Fumarate	0.23 (0.18–0.36)	0.27 (0.19–0.40)
Glucose	4663.9 (4172.3–5417.5)	4925.3 (4503.6–5684.9)^a^
Glutamate	1556.2 (679.8–2785.7)	1342.5 (763.7–2372.1)
Glutamine	4742.5 (2007.9–7169.0)	5073.4 (2219.3–10427.7)
Isoleucine	57.90 (49.81–68.46)	65.02 (55.35–77.95)^b^
Lactate	334.7 (274.2–435.6)	393.3 (323.2–457.6)^b^
Leucine	83.96 (66.70–98.71)	92.19 (76.10–109.92)^a^
Malate	2.37 (1.83–3.66)	2.87 (2.12–3.80)
Pyruvate	9.50 (3.08–21.00)	13.58 (6.00–25.78)
Serine	150.0 (104.9–170.3)	131.9 (106.0–18.8)
Succinate	10.82 (8.44–15.23)	9.11 (8.46–15.15)
Succinyl-CoA	9.79 (6.71–14.00)	13.36 (8.90–18.47)^b^
Valine	99.3 (78.6–125.5)	113.3 (89.6–139.4)^b^

^a^ *p* < 0.05; ^b^*p* < 0.01.

**Table 6:** Spearman's correlation coefficients for age, body mass index, and related metabolites.

	Age		BMI	
	Spearman's rho	*p* value	Spearman's rho	*p* value
3-Hydroxybutirate	0.057	ns	0.022	ns
Aconitate	0.205	0.003	−0.236	0.010
*α*-Ketoglutarate	0.054	ns	−0.031	ns
Alanine	−0.081	ns	0.230	0.013
Aspartate	0.083	ns	0.256	0.005
(Iso)citrate	0.113	ns	0.147	ns
Fumarate	0.220	0.002	−0.085	ns
Glucose	−0.031	ns	0.202	0.029
Glutamate	0.063	ns	−0.032	ns
Glutamine	0.007	ns	0.157	ns
Isoleucine	0.124	ns	0.194	0.036
Lactate	−0.023	ns	0.034	ns
Leucine	−0.005	ns	0.220	0.017
Malate	0.248	<0.001	−0.079	ns
Pyruvate	−0.009	ns	0.103	ns
Serine	0.062	ns	−0.112	ns
Succinate	0.011	ns	−0.145	ns
Succinyl-CoA	0.038	ns	0.134	ns
Valine	−0.122	ns	0.241	0.009

BMI: Body mass index; ns: not significant.

## Data Availability

The data used to support the findings of this study are available from the corresponding author upon request.
